# Single-cell, locus-specific bisulfite sequencing (SLBS) for direct detection of epimutations in DNA methylation patterns

**DOI:** 10.1093/nar/gkv366

**Published:** 2015-04-19

**Authors:** Silvia Gravina, Shireen Ganapathi, Jan Vijg

**Affiliations:** Department of Genetics, Albert Einstein College of Medicine, Bronx, NY 10461, USA

## Abstract

Stochastic epigenetic changes drive biological processes, such as development, aging and disease. Yet, epigenetic information is typically collected from millions of cells, thereby precluding a more precise understanding of cell-to-cell variability and the pathogenic history of epimutations. Here we present a novel procedure for directly detecting epimutations in DNA methylation patterns using single-cell, locus-specific bisulfite sequencing (SLBS). We show that within gene promoter regions of mouse hepatocytes the epimutation rate is two orders of magnitude higher than the mutation rate.

## INTRODUCTION

DNA methylation plays a critical role in the regulation of gene expression and is known to be an essential mechanism for guiding normal cellular development. Numerous studies have implicated aberrant methylation in the etiology of human diseases, including nearly all types of cancer (1). In the past decade, DNA methylation profiling at cytosine-guanine dinucleotides (CpG sites) has gained momentum as an epigenetic approach in basic research and clinical applications.

Most of the techniques currently available can only measure average values obtained from bulk cell populations, requiring at least 30 ng of DNA (i.e., the equivalent of about 6000 cells) (2). Population-wide analyses overlook individual, cell-specific changes, termed epimutations (3) and are unsuited to characterize cellular heterogeneity, which plays such an important role in differentiation and development, stem cell reprogramming, diseases, such as cancer, and aging (4). Developing single-cell approaches for measuring DNA methylation would not only be vital to fully understand individual cell-specific changes and complexity of tissue microenvironments, but also for the analysis of clinical samples, such as circulating tumor cells or needle biopsies, when the amount of material is often limited.

Methods to accurately detect DNA methylation at specific loci typically involve treating DNA with sodium bisulfite (5) or digesting DNA with cytosine methylation-dependent endonucleases (6). While DNA methylation has been assessed in single cells by employing enzymatic digestion in microfluidic devices (7) or pyrosequencing approaches (8), to our knowledge, a simple and inexpensive methodology for detecting epimutations in somatic cells has not been described.

Bisulfite conversion of unmethylated cytosines into uracil is a relatively simple chemical reaction, which has now become a standard in DNA methylation profiling (9). One of the great advantages of this approach lies in having an internal control for conversion rate; indeed, non-CpG cytosines are generally not methylated and, therefore, should be converted by the bisulfite treatment. The key advantage of bisulfite sequencing is accuracy, as the degree of methylation at each cytosine can be quantified with great precision. Others have reported a method for genome-wide single-cell bisulfite sequencing (10). The key advantage of targeted single-cell bisulfite based analyses is that this approach focuses on regions of interest thus greatly reducing the costs.

Here, we present a procedure for single-cell, locus-specific bisulfite sequencing (SLBS) allowing to directly measuring DNA methylation patterns in single cells and estimate epimutation rates. The procedure was extensively validated in fibroblasts, neurons and hepatocytes, analyzing promoter regions of genes known to be either constitutively expressed and hypomethylated or repressed and hypermethylated in these cell populations.

## MATERIALS AND METHODS

### Animals

C57BL/6 mice were obtained from the National Institute on Aging (NIA). All surgical procedures and experimental manipulations were approved by the Ethics Committee for Animal Experiments at the Albert Einstein College of Medicine. Experiments were conducted under the control of the Guidelines for Animal Experimentation. Animals were sacrificed by cervical dislocation.

### Isolation of single mouse embryonic fibroblasts

Mouse embryonic fibroblasts (MEFs) were isolated from embryonic day 13.5 embryos of C57BL/6 mice as described (11). All cultures were maintained in a 3% O_2_ and 5% CO_2_ atmosphere.

### Treatment with 5-aza-2′-deoxycytidine

Cultured MEFs were treated with 1 μM 5-Aza 5-aza-2′-deoxycytidine (5-Aza; Sigma) for 48 and 72 h. After trypsinization, single MEFs were collected under an inverted microscope by hand-held capillaries, deposited in polymerase chain reaction (PCR) tubes and immediately frozen on dry ice and stored at −80°C until needed or immediately bisulfite-converted.

### Isolation of single hepatocytes

Livers in three C57BL/6 mice were perfused with collagenase following the protocol as described (12). Single hepatocytes were collected under an inverted microscope as described for MEFs.

### Single Neuronal Nuclei isolation

Nuclei from mouse brain were first purified and concentrated by centrifugation in a discontinuous density gradient according to the manufacturer's protocol (Sigma). Purified nuclei were tagged by a NeuN antibody that had already been incubated with an anti-mouse IgG Alexa 488 antibody (Invitrogen) following the protocol as described (13). To facilitate visual confirmation that only single nuclei were sorted and not aggregates, nuclei were stained also with DAPI. Individual nuclei were sorted using a MoFlo™ XDP cell sorter (Beckman Coulter) into PCR tubes containing 20 μl of PBS, flash-frozen and stored at −80°C or immediately used for DNA methylation analysis.

### Genomic DNA extraction

DNA from MEF cultures, mouse liver and mouse brain was isolated by phenol/chloroform extraction, as described (14).

### Bisulfite conversion

The bisulfite conversion and recovery of DNA were performed using the EZ DNA Methylation-Direct Kit (Zymo Research). Bisulfite conversion was performed according to the instructions of the supplier with some modifications. First, the bisulfite solution was added to the single or 100 cells together with 2 ng of salmon sperm DNA or tRNA. DNA was denatured for 8 min at 99°C and immediately bisulfite-modified at 64°C for 3.5 h. After conversion, 6 μl of carrier tRNA (1 μg/μl; Qiagen) was added to the DNA binding buffer solution. The addition of carrier RNA enhances the recovery of DNA by preventing the small amount of target nucleic acid present in the sample from being irretrievably bound. Converted DNA was then added to the binding solution/carrier RNA solution and subjected to an in-column purification and desulphonation step followed by two wash steps. Before the final elution, the elution buffer was warmed up at 37°C and then allowed to sit on the column for a few minutes. DNA was eluted in 10 μl of buffer. This protocol was applied to single cells, 100-cell samples and genomic DNA extracted from the mother population or tissue. Immediately upon conversion, converted single-cell or 100-cell DNA was subjected to whole genome amplification. Genomic DNA used as control (starting material 800 ng) was not subjected to whole genome amplification.

### Whole genome amplification

The whole genome amplification of the bisulfite-treated DNA was done using multiple displacement amplification (MDA). The amplification steps were performed using the Whole Bisulfitome kit (Qiagen) according to the instructions of the supplier with some modifications. 29 μl of Master mix and 1 μl of Phi29 DNA polymerase were added to 10 μl of eluted bisulfite converted single-cell DNA (or to 10 μl of 100-cell bisulfite-treated DNA) and incubated as follows: 15 min at 24°C, 16 h at 28°C, followed by a polymerase inactivation step at 95°C for 5 min. The MDA, bisulfite converted DNA was subsequently used as template for bisulfite conversion-specific PCR. MDA amplification generally produces 1 μg of DNA in a final volume of 40 μl.

### Bisulfite conversion-specific PCR

A nested PCR was performed to amplify the target regions. Primers were designed using Sequenom's EpiDesigner software and synthesized by IDT. Sequences of the primers and genomic coordinates of the target regions are available in Supplementary Tables S1 and S2. A T7-promoter tag (5′-CAGTAATACGACTCACTATAGGGAGAAGGCT-3′) was added to the reverse internal PCR primer and a 10-mer-tag sequence (5′-AGGAAGAGAG-3′) was added to the external PCR forward primer to balance the PCR primer length. Approximately 5 μl of bisulfite converted, MDA-amplified DNA was used as a template in the first round of PCR amplification. The reaction was carried out using HotStarTaq Master Mix (Qiagen) in a 50 μl total reaction volume as follows: an initial heat-activation step at 95°C for 15 min, 95°C for 30 s, annealing for 1 min, 72°C for 1 min for a total of 30 cycles ending with a final extension at 72°C for 10 min. The nested PCRs were performed on 5 μl of the first round PCR products. The PCR products were purified using a QIAquick PCR purification kit (Qiagen). Amplicons were Sanger sequenced on the ABI 3730 DNA sequencer or analyzed using the Sequenom EpiTYPER system (Sequenom, Inc) in the Albert Einstein Genomics Core Facility. The latter technique employs base-specific cleavage followed by MALDI-TOF mass spectrometry in which the size ratio of the cleaved products provides quantitative methylation estimates for CpG sites within a target region. Each EpiTYPER run was performed in duplicate. Results were analyzed using the EpiTYPER software.

## RESULTS

**Figure 1.**
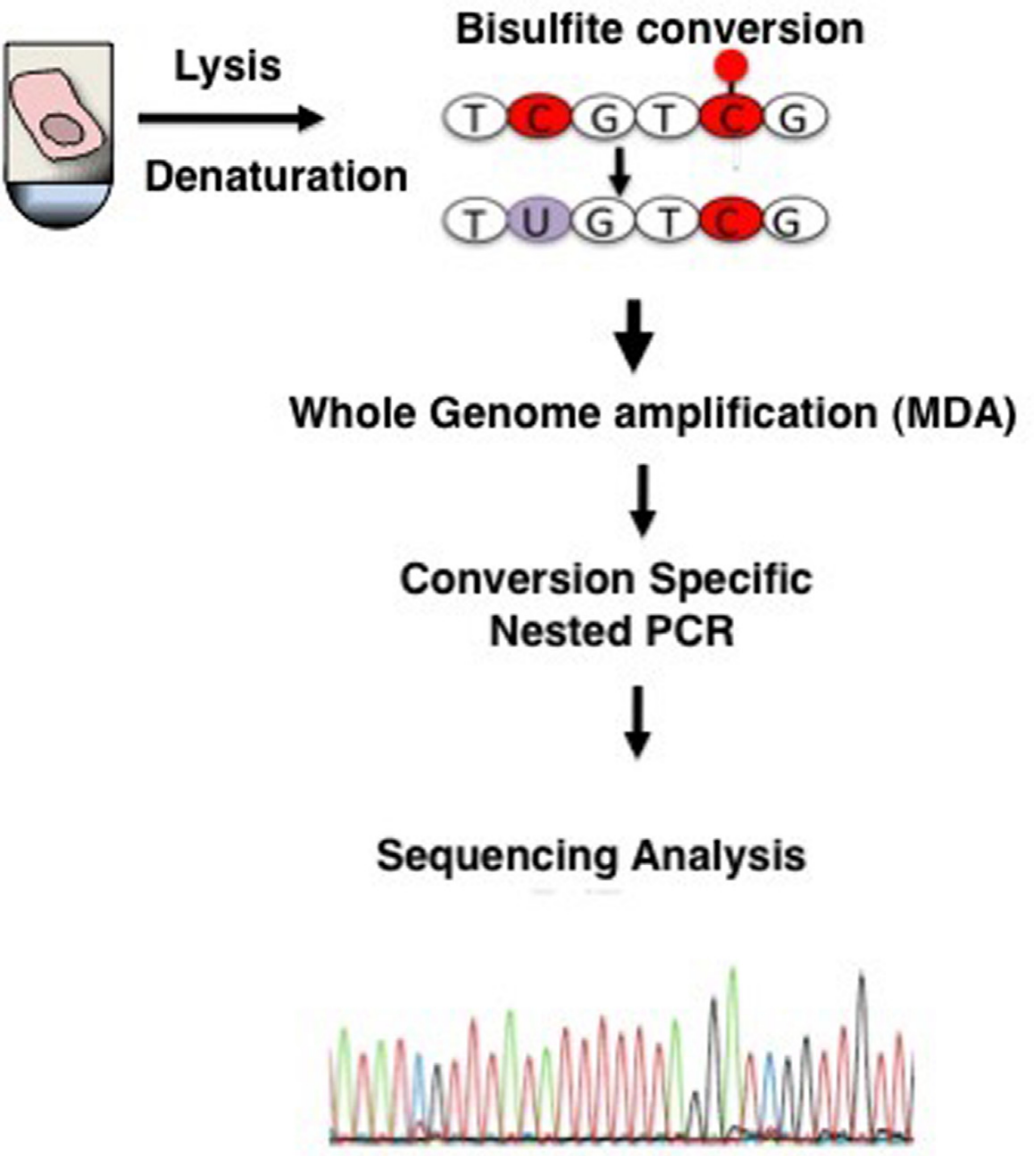
Schematic depiction of single-cell DNA methylation analysis. Single cells are lysed and treated with bisulfite to convert unmethylated cytosines into uracil. After bisulfite treatment, DNA is immediately whole-genome amplified by MDA. DNA methylation patterns are analyzed in a locus-specific way using PCR amplification with primers specific for cytosine-converted DNA.

To develop and test SLBS, single cells were collected from populations of MEFs under an inverted microscope by hand-held capillaries, and either frozen or immediately subjected to heat DNA denaturation, followed by bisulfite treatment. The converted DNA was subsequently subjected to whole genome amplification using MDA, based on phi29 DNA polymerase and random hexamer primers in an isothermal reaction. The bisulfite-converted, amplified material was then used as template for conversion-specific PCR, targeting regions of interests. Purified PCR products were then subjected to sequencing analysis. The procedure is schematically depicted in Figure 1.

One of the major limitations of bisulfite sequencing for DNA methylation analysis is its propensity for DNA degradation due to partial, acid-catalyzed depurination (15). Consequently, a high proportion of the template DNA is too fragmented to be analyzed. In addition, if the treatment is too harsh or prolonged, a small portion of 5-methylcytosines may also be converted to uracil, resulting in falsely concluding the absence of methylation (16). Milder treatment, conversely, results in incomplete conversion and false positives.

Therefore, in our protocol, we had to find a balance between under- and over-treatment, trying to achieve both high sensitivity and high specificity in detecting epimutations. To accomplish this we optimized bisulfite conversion by testing promoter regions of genes known to be either hyper- or hypo-methylated in bulk MEFs, i.e., 141 bp of the promoter region of the *Nfe2l2* stress response gene, which is constitutively expressed and hypomethylated in MEFs (17), and 180 bp of the promoter region of the transcription factor *Oct4* (also known as Pou5f1), which is both methylated and non-expressed in differentiated cells (18). PCR primers were designed to amplify only converted sequences, that is, sequences in which non-methylated cytosines are replaced by thymines. To increase specificity, we used a nested PCR approach. As positive controls we used collections of 100 MEFs, bisulfite-treated and MDA-amplified in the same way as the single cells, as well as 800 ng of bisulfite-treated, unamplified DNA from the same MEF population. Non-bisulfite-treated, non-amplified, genomic DNA served as negative control to verify PCR specificity for only fully converted DNA.

**Figure 2.**
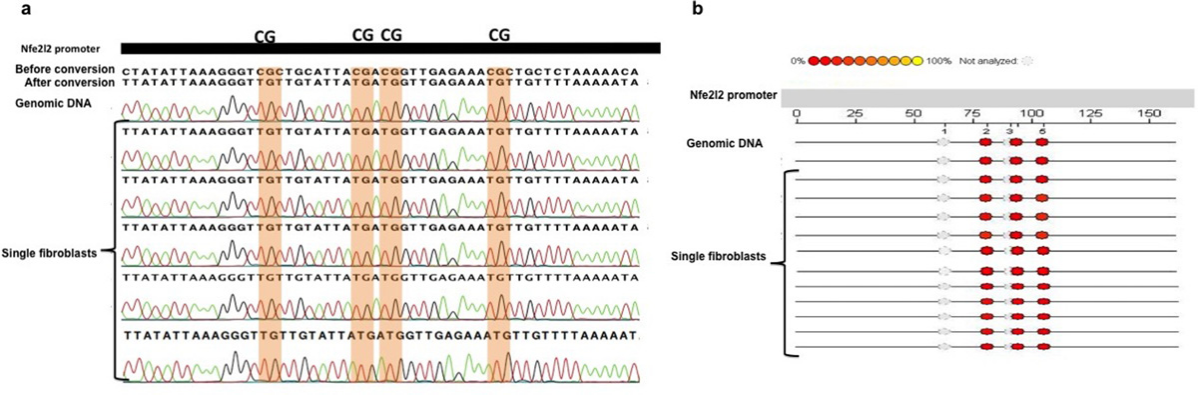
(**a**) DNA methylation profile of a 180 bp fragment of the *Nfe2l2* promoter in single fibroblasts analyzed using Sanger Sequencing. (**b**) DNA methylation profile of a 180 bp fragment of the *Nfe2l2* promoter in single fibroblasts analyzed using Sequenom's EpiTYPER System. The EpiGram is a graphical representation of methylation ratios found in each sample for the amplicon studied. Each sample's nucleotide sequence is displayed as a series of individual CpGs, which are color-coded columns on the same line. The color within the column denotes the level of methylation found at this particular site in the selected sample. The color spectrum ranges from red (0% methylated) CpG units to yellow (100% methylated) CpG units. Gray dots denote not analyzable CpG sites.

In non-pluripotent cell types, cytosines not followed by guanines are methylated only very rarely (19), and this was used as an indication of bisulfite conversion efficiency, calculating the C to T conversion rate for all cytosine bases other than those in CpG dinucleotides. Initial results indicated that bisulfite conversion of cytosines at relatively low temperatures (i.e., 37°C) is generally incomplete (∼80% conversion rate; data not shown). An optimal degradation versus conversion ratio was obtained by incubating DNA for 3.5 h at 64°C. Under these conditions we obtained full conversion of unmethylated cytosines with minimal degradation. However, we also observed occasional conversion of methylated CpGs in the single cells but not in the controls. We reasoned that this was likely due to the bisulfite treatment conditions being too harsh for the only ∼5 pg of DNA in a single cell, but not for the 100-cell sample or 800 ng total genomic DNA. Therefore, we modified our protocol by adding 2 ng (i.e., the equivalent of a few hundred cells) of salmon sperm DNA or tRNA as a “competitor” to the single cell DNA in the conversion reaction in order to reduce the over-exposure of the single-cell DNA to the bisulfite. Under these conditions we could greatly reduce conversion of methylated cytosines while maintaining high conversion of unmethylated cytosines. With the newly modified protocol, nested PCR for the *Nfe2l2* promoter resulted in a product in about 40% and 99% of the time in single cells and 100-cell samples, respectively (Supplementary Figure S1a and b); similar results were obtained for *Oct4* (not shown). Examples of Sanger sequencing results for the *Nfe2l2* promoter in single fibroblasts are shown in Figure 2a. To further confirm these results in an independent experiment, the *Nfe2l2* promoter DNA methylation patterns were quantified using the Sequenom EpiTYPER System (Figure 2b). In order to detect epimutations, i.e., random changes in methylation status of single CpG sites (as a consequence of, for example, errors in *de novo* or maintenance methylation), we compared DNA methylation profiles between the single MEFs and the bulk. No epimutations were detected in the case of MEFs.

**Figure 3.**
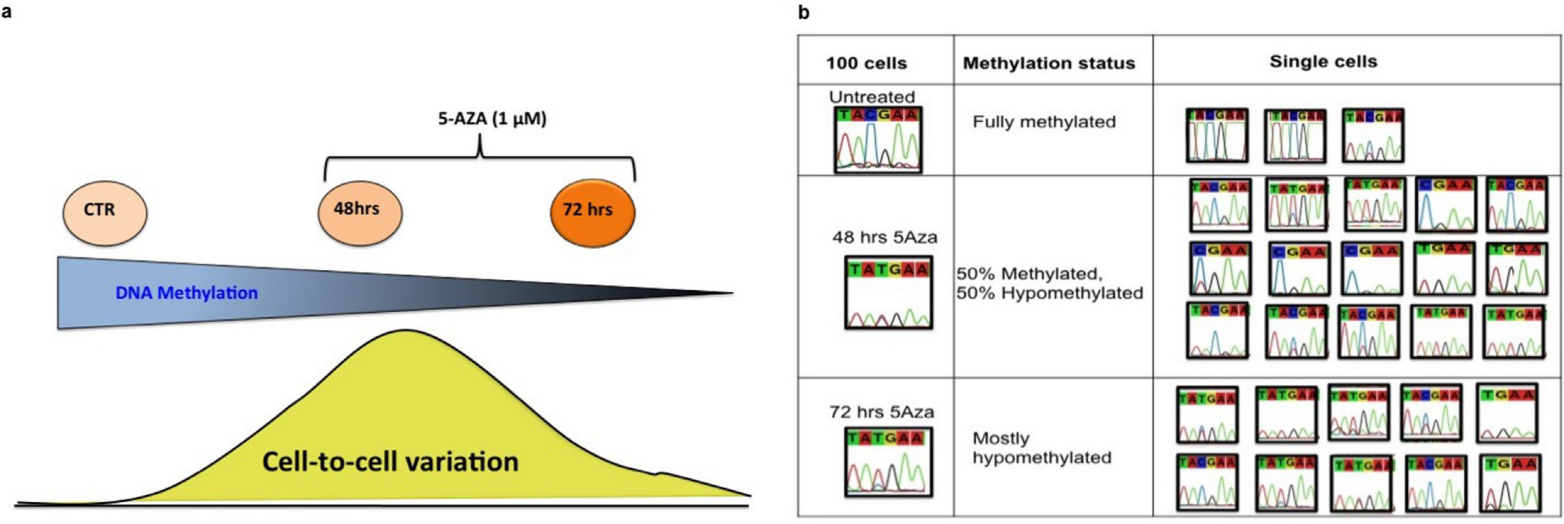
(**a**) A schematic depiction of the 5-Aza-time course experiment in single fibroblasts. (**b**) Representative example of DNA methylation profiles of *Oct4*-specific CpG sites in MEFs treated with 1 μM 5-Aza for 48 and 72 h. As expected, *Oct4* CpG sites in the untreated single fibroblasts as well as in the 100-cell control were mostly methylated (top panel), while after 72 h of treatment the *Oct4* promoter was mostly demethylated in the 100-cell control as well in the single fibroblasts (lower panel). The recovery of both C and T alleles, particularly evident after 48 h of treatment (middle panel), could be interpreted as a 5-Aza-induced hemi-demethylation, caused by incomplete demethylation possibly due to failure of the covalent 5-Aza/Dnmt1 binding on one allele.

To further validate SLBS, we designed an experiment that would conclusively show that the method was able to detect cell-to-cell heterogeneity in DNA methylation patterns. For this purpose we performed a time course experiment with MEFs treated with 5-azacytidine (5-Aza), schematically depicted in Figure 3a. 5-Aza prevents methylation at CpG sites in newly synthesized DNA through covalent binding to Dnmt1 (20,21). Hence, when analyzing a genomic fragment fully methylated in bulk cells we would expect a stochastic increase in cells that become hypomethylated at CpG's in that fragment until after several rounds of cell division the fragment would be fully demethylated. Cultured cells were treated with 1 μM 5-Aza for 48 and 72 h, after which ten single cells as well as 100-cell controls from each treatment group were subjected to the single-cell bisulfite procedure. We then analyzed the promoter region of *Oct4* and showed that, as expected, CpG sites in the untreated single fibroblasts as well as in the 100-cell control were mostly methylated (Figure 3b, upper panel). After 72 h of treatment the *Oct4* promoter was mostly demethylated, both in single fibroblasts and in the 100-cell control sample (Figure 3b, lower panel). After 48 h of treatment, DNA demethylation was incomplete as could be concluded from the 100-cell sample, which showed about 50% demethylation (i.e., a mixed C and T peak). This suggested the presence of a mixed population of unmethylated and methylated CpG sites. When we analyzed the *Oct4* promoter in single fibroblasts upon 48 h of treatment, we found a mixed population comprising either methylated or unmethylated cytosines. Some cells showed either a C or a T peak (Figure 3b, middle panel). However, within the same cell(s), we also observed the recovery of both (C and T) alleles (Figure 3b, middle panel), which may represent 5-Aza-induced hemi-demethylation events, possibly due to failure of 5-Aza to block methylation on one allele in the cell.

**Figure 4.**
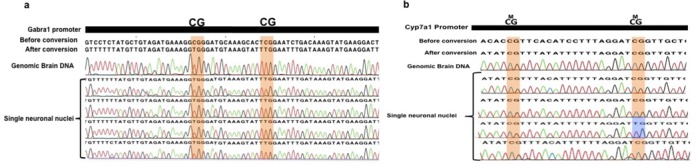
(**a**) DNA methylation profiling of *Gabra1* in single neuronal nuclei. Cytosines of CpG dinucleotides are highlighted in orange. (**b**) *Cyp71a* promoter in single neuronal nuclei. Cytosines of CpG dinucleotides are highlighted in orange. Epimutations are highlighted in blue.

After having validated SLBS we wished to demonstrate its usefulness in analyzing other cell types *in vivo*. First, we applied the method to single neurons. Because the entanglement of neurons with axons makes the manual capillary picking challenging, we opted for isolating neuronal nuclei. Nuclei from whole mouse brain were isolated using a discontinuous sucrose gradient, stained with the neuron-specific monoclonal antibody NeuN, and subsequently with DAPI, and deposited in PCR tubes using FACS. Promoter regions were selected for genes either constitutively expressed and hypomethylated *(Gabra1)*, or repressed and hypermethylated (*Cyp71a*) in brain. Figure 4a and b show Sanger sequencing results for 4 single neuronal nuclei for *Gabra1 and for Cyp71a*. By comparing DNA methylation patterns in single neuronal nuclei with the bulk, we were able to detect one (demethylating) epimutation event (out of a total of 8 CpG sites analyzed) in the *Cyp71a* promoter (highlighted in blue in Figure 4b). In the *Gabra1* promoter, we did not detect any (methylating) events. Of note, we also observed a non-conversion event at two C (AT) sites in the *Cyp71a* promoter (Figure 4b). This may represent a non-CpG methylation event, rather than incomplete bisulfite conversion. Indeed, others have shown that in the brain, CHH methylation is the most common methylation event after the CpG one (22).

**Figure 5.**
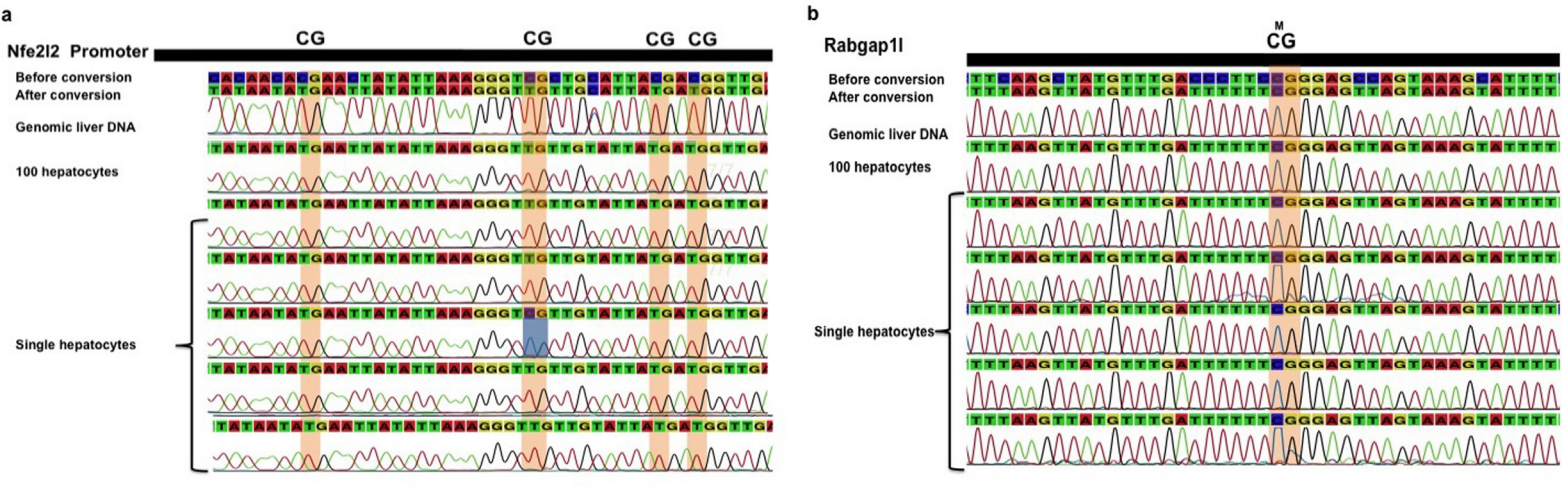
(**a**) DNA methylation profile of the *Nfe2l2* promoter in single hepatocytes. Cytosines of CpG dinucleotides are highlighted in orange. (**b**) DNA methylation profile of Rabgap1l (intragenic region) in single hepatocytes. Cytosines of CpG dinucleotides are highlighted in orange. Epimutations are highlighted in blue.

Next, we applied SLBS to mouse hepatocytes. As we did for fibroblasts and neurons, we selected regions of genes either constitutively expressed and hypomethylated, or repressed and hypermethylated in liver. Initially, we looked at four single hepatocytes and two genes *Nfe2l2* and *Rabgap1l*. Figure 5a and b show Sanger sequencing results for four single hepatocytes for *Nfe2l2* and *Rabgap1l*. In this specific experiment, a methylating event was detected in the *Nfe2l2* promoter (highlighted in blue in Figure 5a) while In the *Rabgap1l gene*, we did not detect any demethylating events.

Finally, while the occasional single-cell methylation or demethylation events suggests the occurrence of epimutations, not enough cells were analyzed to estimate an epimutation frequency. We now applied SLBS for directly measuring, for the first time, epimutation rates at gene promoters in mouse liver *in vivo* by analyzing 5 additional genes and a total of 206 hepatocytes from different individual mice. While estimates of DNA methylation accuracy have been reported (23,24), it has never been possible to directly determine such epimutations. In addition to the previously studied *Nfe2l2* and *Rabgap1l*, we selected regions of genes either constitutively expressed and hypomethylated, or repressed and hypermethylated in liver. Table 1 summarizes the results obtained for a total of 601 interrogated CpG sites. Notably, out of a total of 3296 non-CpG cytosines, 3279 were converted into uracil (and subsequently thymine), a conversion rate of well over 99.4%.

**Table 1.** Locus-specific bisulfite sequencing of single hepatocytes

				Total epimutations		
Gene region	Number of CpGs per region	Total cells analyzed	Total CpGs for all the cells analyzed	Demethylating	Methylating	Conversion of unmethylated non-CpG cytosines
*Oct4* (region1)	2	17	34	6		290/293
*Oct 4* (region 2)	4	12	48	3		230/230
L1 (Chr18)	2	33	66	3		48/48
*Gabra-1*	3	66	198			724/726
*Cyp71a*	2	24	48		4	733/740
*Nfe2l2*	4	34	136		6	428/429
*Rabgap1l*	4	11	44	3		692/ 695
*Dpf1*	3	9	27	1		151/152
		206	601	16 (Demethylating epimutation Rate: 2.7%)	10 (Methylating epimutation rate: 1.6%)	3279/3296 (Conversion rate: 99.4%)

To identify differential methylation and demethylation events we compared methylation patterns in the single cells with those for the whole liver tissue. The epimutation rate is then the number of altered CpG methylation sites versus the total number of CpGs analyzed. In this way we found that the rate of epimutational loss of methyl groups was 2.7% with a rate of epimutational gain of 1.6% (Table 1).

Of note, because the cells studied are diploid (or may be even polyploid in the case of hepatocytes (12), we are targeting at least two alleles per cell. However, MDA has allelic bias and as we (25,26), and others (27) have shown, in about 10% of cases amplification occurs from one allele only, which is randomly distributed across the genome. Therefore, we can never rule out that a single peak in the Sanger sequence (see representative examples in Supplementary Figure S2a and b) truly reflects homozygous methylation status or is due to allelic bias of the amplification. In calculating epimutation rates, unless two peaks could clearly be distinguished in the Sanger sequence (see representative example in Supplementary Figure S2c), we made the assumption that only one allele is represented. In addition, while for the demethylating epimutations we cannot rule out the possibility that these represent accidental conversions of methylated cytosines rather than genuine demethylating events, for the methylation mutations we verified that their rate was significantly higher than the non-conversion rate of unmethylated cytosines (Fisher's exact test, *P* < 0.05).

The frequency of methylating and demethylating epimutation events is about two orders of magnitude higher than the general mutation frequency of DNA sequences in somatic mouse cells (14). While to our knowledge this has never before reported for mammalian cells, these results are in accordance with recent results suggesting that spontaneous transgenerational epigenetic changes in the Arabidopsis thaliana methylome are three orders of magnitude more frequent than DNA mutations (28,29).

## DISCUSSION

Thus far, virtually all epigenomic information available to us is derived from measurements on mixtures of large numbers of cells, thereby precluding a more precise understanding of cell-to-cell variability and the pathogenic history of epimutations. While there has been progress with genome-wide approaches, and a few groups (7,8) described methodologies to assess DNA methylation in single cells, a simple and inexpensive methodology for detecting epimutations in somatic cells has not been described.

Here we presented a method for single-cell, locus specific bisulfite sequencing (SLBS), which allows to accurately measure epimutation rates. One of the great advantages of bisulfite based approaches is the ready availability of an internal control for conversion rate; cytosines that are not followed by guanine are not methylated and, therefore, should be converted in uracil by the bisulfite treatment. A key advantage of bisulfite-based methods is accuracy, as the degree of methylation at each cytosine can be quantified with great precision.

Our SLBS procedure was extensively validated in fibroblasts, neurons and hepatocytes, analyzing promoter regions of genes known to be either constitutively expressed and hypomethylated or repressed and hypermethylated in these cell populations. By comparing DNA methylation patterns in single cells with those in the tissue from which they were derived we were able to directly measure the “epimutation frequency” within promoter regions, which appeared to be two orders of magnitude higher than somatic mutation frequencies as obtained in the past from reporter gene studies (14). In this respect, it would be of interest to apply SLBS to study epimutations in human tumors to complement the DNA sequence mutations available through The Cancer Genome Atlas (TCGA).

SLBS can be applied not only to basic research to study phenotypic diversity within organs and tissues in relation to disease states, but also to improve diagnostic and prognostic assays that sample very small numbers of cells from affected areas of diseased tissues. One major clinical application is to assess DNA methylation patterns in promoter regions of tumor suppressor genes in circulating tumor cells (30). For diagnostic purposes, our procedure could be implemented in a high throughput approach, for example, by coupling SLBS with microfluidics-based multiplex PCR (31,32), to simultaneously amplify and analyze a large number of CpG sites at very low cost.

We anticipate that SLBS will contribute to the current shift in the molecular biosciences from average endpoints toward the description of cell populations, tissues and organs through their individual parts at single-cell resolution.

## SUPPLEMENTARY DATA

Supplementary Data are available at NAR Online.

SUPPLEMENTARY DATA

## References

[1] Jones P.A. (2002). DNA methylation and cancer. Oncogene.

[2] Gu H., Bock C., Mikkelsen T.S., Jager N., Smith Z.D., Tomazou E., Gnirke A., Lander E.S., Meissner A. (2010). Genome-scale DNA methylation mapping of clinical samples at single-nucleotide resolution. Nat. Methods.

[3] Jeggo P.A., Holliday R. (1986). Azacytidine-induced reactivation of a DNA repair gene in Chinese hamster ovary cells. Mol. Cell. Biol..

[4] Egger G., Liang G., Aparicio A., Jones P.A. (2004). Epigenetics in human disease and prospects for epigenetic therapy. Nature.

[5] Frommer M., McDonald L.E., Millar D.S., Collis C.M., Watt F., Grigg G.W., Molloy P.L., Paul C.L. (1992). A genomic sequencing protocol that yields a positive display of 5-methylcytosine residues in individual DNA strands. Proc. Natl. Acad. Sci. U.S.A..

[6] Khulan B., Thompson R.F., Ye K., Fazzari M.J., Suzuki M., Stasiek E., Figueroa M.E., Glass J.L., Chen Q., Montagna C. (2006). Comparative isoschizomer profiling of cytosine methylation: the HELP assay. Genome Res..

[7] Kantlehner M., Kirchner R., Hartmann P., Ellwart J.W., Alunni-Fabbroni M., Schumacher A. (2011). A high-throughput DNA methylation analysis of a single cell. Nucleic Acids Res..

[8] El Hajj N., Trapphoff T., Linke M., May A., Hansmann T., Kuhtz J., Reifenberg K., Heinzmann J., Niemann H., Daser A. (2011). Limiting dilution bisulfite (pyro)sequencing reveals parent-specific methylation patterns in single early mouse embryos and bovine oocytes. Epigenetics.

[9] Fraga M.F., Esteller M. (2002). DNA methylation: a profile of methods and applications. Biotechniques.

[10] Smallwood S.A., Lee H.J., Angermueller C., Krueger F., Saadeh H., Peat J., Andrews S.R., Stegle O., Reik W., Kelsey G. (2014). Single-cell genome-wide bisulfite sequencing for assessing epigenetic heterogeneity. Nat. Methods.

[11] Garcia A.M., Busuttil R.A., Rodriguez A., Cabrera C., Lundell M., Dollé M.E., Vijg J. (2007). Detection and analysis of somatic mutations at a lacZ reporter locus in higher organisms: application to Mus musculus and Drosophila melanogaster. Methods Mol. Biol..

[12] Faggioli F., Sacco M.G., Susani L., Montagna C., Vezzoni P. (2008). Cell fusion is a physiological process in mouse liver. Hepatology.

[13] Matevossian A., Akbarian S. (2008). Neuronal nuclei isolation from human postmortem brain tissue. J. Vis. Exp.

[14] Busuttil R.A., Garcia A.M., Reddick R.L., Dollé M.E., Calder R.B., Nelson J.F., Vijg J. (2007). Intra-organ variation in age-related mutation accumulation in the mouse. PLoS One.

[15] Raizis A.M., Schmitt F., Jost J.P. (1995). A bisulfite method of 5-methylcytosine mapping that minimizes template degradation. Anal. Biochem.

[16] Genereux D.P., Johnson W.C., Burden A.F., Stoger R., Laird C.D. (2008). Errors in the bisulfite conversion of DNA: modulating inappropriate- and failed-conversion frequencies. Nucleic Acids Res..

[17] Hayes J.D., Chanas S.A., Henderson C.J., McMahon M., Sun C., Moffat G.J., Wolf C.R., Yamamoto M. (2000). The Nrf2 transcription factor contributes both to the basal expression of glutathione S-transferases in mouse liver and to their induction by the chemopreventive synthetic antioxidants, butylated hydroxyanisole and ethoxyquin. Biochem. Soc. Trans..

[18] Loh Y.H., Wu Q., Chew J.L., Vega V.B., Zhang W., Chen X., Bourque G., George J., Leong B., Liu J. (2006). The Oct4 and Nanog transcription network regulates pluripotency in mouse embryonic stem cells. Nat. Genet..

[19] Ziller M.J., Muller F., Liao J., Zhang Y., Gu H., Bock C., Boyle P., Epstein C.B., Bernstein B.E., Lengauer T. (2011). Genomic distribution and inter-sample variation of non-CpG methylation across human cell types. PLoS Genet..

[20] Palii S.S., Van Emburgh B.O., Sankpal U.T., Brown K.D., Robertson K.D. (2008). DNA methylation inhibitor 5-Aza-2′-deoxycytidine induces reversible genome-wide DNA damage that is distinctly influenced by DNA methyltransferases 1 and 3B. Mol. Cell. Biol..

[21] Maslov A.Y., Lee M., Gundry M., Gravina S., Strogonova N., Tazearslan C., Bendebury A., Suh Y., Vijg J. (2012). 5-Aza-2′-deoxycytidine-induced genome rearrangements are mediated by DNMT1. Oncogene.

[22] Guo J.U., Su Y., Shin J.H., Shin J., Li H., Xie B., Zhong C., Hu S., Le T., Fan G. (2014). Distribution, recognition and regulation of non-CpG methylation in the adult mammalian brain. Nat. Neurosci..

[23] Ushijima T., Watanabe N., Okochi E., Kaneda A., Sugimura T., Miyamoto K. (2003). Fidelity of the methylation pattern and its variation in the genome. Genome Res..

[24] Laird C.D., Pleasant N.D., Clark A.D., Sneeden J.L., Hassan K.M., Manley N.C., Vary J.C., Morgan T., Hansen R.S., Stoger R. (2004). Hairpin-bisulfite PCR: assessing epigenetic methylation patterns on complementary strands of individual DNA molecules. Proc. Natl. Acad. Sci. U.S.A..

[25] Gundry M., Li W., Maqbool S.B., Vijg J. (2012). Direct, genome-wide assessment of DNA mutations in single cells. Nucleic Acids Res..

[26] Li W., Calder B., Mar J.C., Vijg Jan (2015). Single-cell transcriptogenomics reveals transcriptional exclusion of ENU-mutated alleles. Mutat. Res..

[27] Evrony G.D., Cai X., Lee E., Hills L.B., Elhosary P.C., Lehmann H.S., Parker J.J., Atabay K.D., Gilmore E.C., Poduri A. (2012). Single-neuron sequencing analysis of L1 retrotransposition and somatic mutation in the human brain. Cell.

[28] Schmitz R.J., Schultz M.D., Lewsey M.G., O'Malley R.C., Urich M.A., Libiger O., Schork N.J., Ecker J.R. (2011). Transgenerational epigenetic instability is a source of novel methylation variants. Science.

[29] Becker C., Hagmann J., Muller J., Koenig D., Stegle O., Borgwardt K., Weigel D. (2011). Spontaneous epigenetic variation in the Arabidopsis thaliana methylome. Nature.

[30] Pantel K., Alix-Panabieres C. (2010). Circulating tumour cells in cancer patients: challenges and perspectives. Trends Mol. Med..

[31] Ying L., Wang Q. (2013). Microfluidic chip-based technologies: emerging platforms for cancer diagnosis. BMC Biotechnol..

[32] Kalisky T., Quake S.R. (2011). Single-cell genomics. Nat. Methods.

