# Supplementary material for: Single-cell, locus-specific bisulfite sequencing (SLBS) for direct detection of epimutations in DNA methylation patterns
Source: Nucleic Acids Res. 2015 Apr 19;43(14):e93. doi: 10.1093/nar/gkv366 (PMC4538804; doi:10.1093/nar/gkv366)
Supplement: SUPPLEMENTARY DATA [file supp_43_14_e93__index.html]

Single-cell, locus-specific bisulfite sequencing (SLBS) for direct detection of epimutations in DNA methylation patterns — SUPPLEMENTARY DATA 

# Single-cell, locus-specific bisulfite sequencing (SLBS) for direct detection of epimutations in DNA methylation patterns

## SUPPLEMENTARY DATA

**Files in this Data Supplement:**

- SUPPLEMENTARY DATA
