# Supplementary material for: Single-cell, locus-specific bisulfite sequencing (SLBS) for direct detection of epimutations in DNA methylation patterns
Source: Nucleic Acids Res. 2015 Apr 19;43(14):e93. doi: 10.1093/nar/gkv366 (PMC4538804; doi:10.1093/nar/gkv366)
Supplement: SUPPLEMENTARY DATA [file supp_gkv366_nar-03614-met-g-2014-File007.pdf]

Supplementary Material

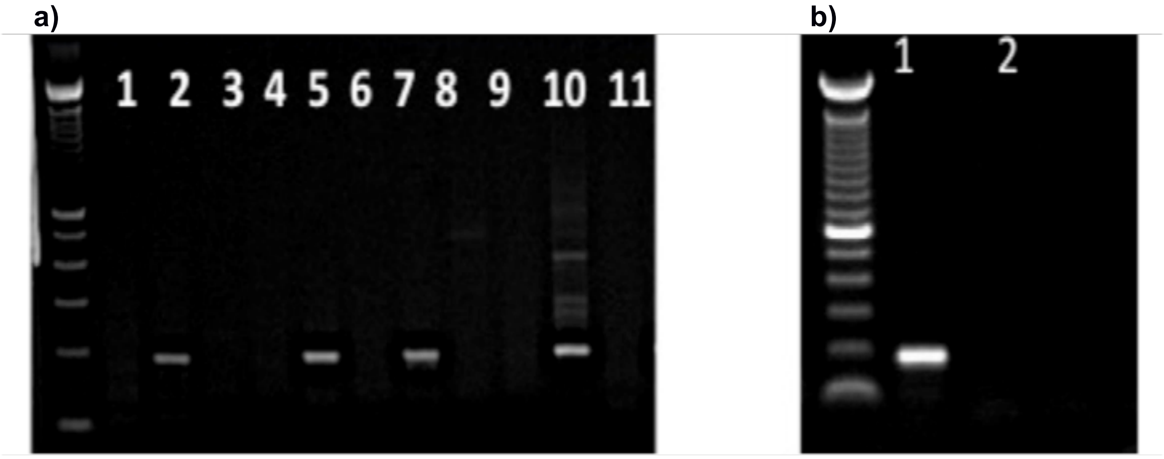

Supplementary Figure 1. Representative examples of conversion-specific, nested PCR of the *Nfe212* promoter from bisulfite converted single MEFs (Supplementary Fig.1a-1-9), from a pool of 100 cells (Supplementary Fig.1a-10) and from a negative control, i.e., water. To verify the specificity of the PCR for amplifying only converted DNA, 800 ng of unamplified, bisulfite-treated (Supplementary Fig.1b-1) and untreated (Supplementary Fig.1b-2), genomic DNA were subjected to the same nested PCR.

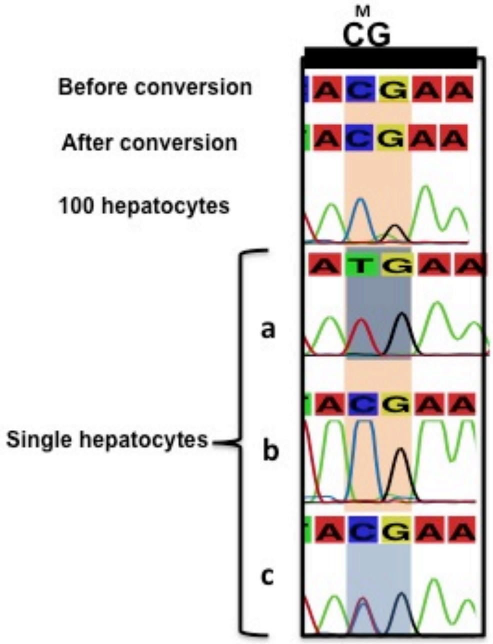

Supplementary Figure 2. Representative examples of a homozygously demethylating event (a), with only the thymine peak present in the Sanger sequence and a hemi-demethylating event (c) with both alleles (C/T) being represented. In (b) both alleles remain methylated. Of note, for homozygous demethylation we can never tell if this is not due to allele-specific amplification (see main text).

Supplementary Table 1. Primer sequences used for locus specific assays

Gene name	Primer sequence
Nfe212-FW External	5’-AGTGGTATAGTTTTTAGTTTGTGGAGAGT -3’
Nfe212-RW External	5’-CCACCCAAAACCCAACAAAC-3’
Nfe212-FW Internal	5’-AGGAAGAGAGAGTTATGAAGTAGTAGTAAAAA-3’
Nfe212-RW Internal	5’-CAGTAATACGACTCACTATAGGGAGAAGGCTAATATAATCTCATAAAACCCCAC-3’
Cyp71a-FW External	5’-GAGTGAATTTTTAAGTTATGGTTGTTT-3’
Cyp71a-RW External	5’-AACAAACAAAAACTTTCCATCCTAA-3’
Cyp71a-FW Internal	5’-AGGAAGAGAGTTGTTTAGAAGATGAGTGTTGGGAG-3’
Cyp71a-RW Internal	5’-CAGTAATACGACTCACTATAGGGAGAAGGCTCAAACAAAAACTTTCCATCCTAA CTT-3’
Rab-FW External	5’-AGTGATTGGGAGTTTTGTAGTTTTGTA-3’
Rab-RW External	5’-CAGTAATACGACTCACTATAGGGAGAAGGCTAAAAAAAACAATTACTCTAACTTTCA CA-3’
Rab-FW Internal	5’-AGGAAGAGAGGTGGTAAAGTGTTTGTTTATAGAAGGG-3’
Rab-RW Internal	5’CAGTAATACGACTCACTATAGGGAGAAGGCTAAATAAACCTCCAAATCTACTACCC-3’
OCT4-reg2-FW External	5’-GTTTTGGATATGGGTTGAAATATTG-3’
OCT4-reg2-RW External	5’-CCACCCTCTAACCTTAACCTCTAAC-3’
OCT4-reg2-FW Internal	5’-AGGAAGAGAGGATATGGGTTGAAATATTGGGTTTAT-3’
OCT4-reg2-RW Internal	5’-CAGTAATACGACTCACTATAGGGAGAAGGCTAATCCTCTCACCCCTACCTTAAAT-3’
OCT4-reg1- FW External	5’-AGGAAGGTTGAAAATGAAGGTTTTTT-3’
OCT4-reg1-RW External/Internal	5’-CAGTAATACGACTCACTATAGGGAGAAGGCTCCACCCTCTAACCTTAACCTCTAAC-3’
OCT4-reg1-FW Internal	5’-AGGAAGAGAGAAGGTTGAAAATGAAGGTTTTTT G-3’
L1chr18-FW External	5’-TATAGGGGAATGTTAGGGTTAAGAAG-3’
L1chr18-RW External	5’-CAAAACAATACCACCTCAAAACTA CT-3’
L1chr18-FW Internal	5’-AGGAAGAGAGATAGGGGAATGTTAGGGTTAAGAAG-3’
L1chr18-RW Internal	5’-CAGTAATACGACTCACTATAGGGAGAAGGCTCCTAAAACCTAAAAATTACAAAC ATAATT-3’
Dpf1-FW External	5’- TTTTTGGGATTTAGTTTTTGTTTTA -3’
Dpf1-RW External/Internal	5’-CAGTAATACGACTCACTATAGGGAGAAGGCTACAAAAACCTTTTCCTTCAAATA CC-3’
Dpf1-FW Internal	5’-AGGAAGAGAGAGGATTAGTTGTTGTTTTGTGATGA-3’
Gabra1-FW External	5’-TTTTTAGGGAAGGTAAGAAAAGAGGAT-3’
Gabra1-RW External/Internal	5’- CAGTAATACGACTCACTATAGGGAGAAGGCTCCTGAAAAAGCACAGAGACTCC -3’
Gabra1-FW Internal	5’-AGGAAGAGAGTGGAGTTTAAGGTAAAGGTATGTTTT-3’

Supplementary Table 2. Genomic coordinates of targeted regions

Gene name	Chr, accession number, sequence coordinates
Nfe212 External PCR	CHR2, NC_000068.7, (75705726; 75705387)
Nfe212 Internal PCR	CHR2, NC_000068.7, (75705634; 75705496)
Cyp71a External PCR	CHR4, AC_000026.1, (6195665; 6195374)
Cyp71a Internal PCR	CHR4, AC_000026.1, (6195645; 6195380)
Rab External PCR	CHR1, NC_000067.6, (160308632; 160309119)
Rab Internal PCR	CHR1, NC_000067.6, (160308665; 160309072)
Oct4/region1 External PCR	CHR 17, AC_000039.1 (39020533; 39020963)
Oct4/region1 Internal PCR	CHR 17, AC_000039.1 (39020765; 39020963)
Oct4/region2 External PCR	CHR 17, AC_000039.1 (39020524; 39020963)
Oct4/region2 Internal PCR	CHR 17, AC_000039.1 (39020537; 39020783)
L1chr18 External PCR	CHR18, NC_000084, (35090757; 35091027)
L1chr18 Internal PCR	CHR18, NC_000084, (35090922; 35091027)
Dpf1 External PCR	CHR7, AC_000029, (23886514; 23886893)
Dpf1 Internal PCR	CHR7, AC_000029, (23886603; 23886893)
Gabra External PCR	CHR11, NC_000077, (42183377; 42183131)
Gabra Internal PCR	CHR11, NC_000077, (42183267; 42183131)
